# Chemical Breath

**DOI:** 10.1007/978-3-030-57081-1_3

**Published:** 2020-10-14

**Authors:** Anita Hardon

**Affiliations:** University of Amsterdam, Amsterdam, Noord-Holland the Netherlands

## Abstract

*Chemical Breath* presents two focused ethnographies that look at the relationship between young people and the inhaling of tobacco and synthetic cannabinoids. The first comes from a group of young people in Paris who smoke electronic cigarettes (e-cigarettes), who refer to themselves as “*vapoteurs*,” and the second comes from a group of young people in Makassar who smoke synthetic cannabinoids. The young people partaking in these popular practices value the social bonding they experience; they are also bombarded with social media messages encouraging the use of these products. And both face harms that may increase the precariousness of their lives: the Makassarian youth face imprisonment if discovered, and the health consequences of these synthetics are not fully understood. Similarly, the Parisian youth also risk lung damage, as vaping, while advertised as “safer” and sought out as a means to reduce the harms associated with cigarette smoke, exposes consumers to chemicals that either are understudied or known to be threats to health. The chapter concludes by pointing how these young people’s lives would benefit from sensible government regulation.

James Monsees and Adam Bowen, two product design students at Stanford University, California, met each other while taking smoke breaks 15 years ago. Their failed attempts to quit inspired them to develop a vaping device that could help people stop smoking. Studying at a Silicon Valley university that encourages entrepreneurship, they soon set up their own company, Ploom, Inc. (later Pax Labs), where they designed a new slick device that worked by heating tobacco in a small, very hot oven, creating a vapor that could be inhaled. Monsees, CEO of Ploom, stated that the product offered a “premium luxury vape experience” (Abrahamian 2014). By 2015, the company had developed JUUL, a slim, rectangular vaping device, which can be charged via a USB port. Monsees points out that the combustion mechanisms of the e-cigarette creates a physiological “buzz,” similar to the sharp peak of nicotine after the first puff of a cigarette (Tiku 2019).

The focused ethnographies presented in this chapter show how young people inhale tobacco and new kinds of synthetic cannabinoids in the form of e-cigarettes or vape devices. Our team found that they turn to these new products in groups, exchanging experiences and trying out new tastes. The inherently social character of these collaborative experiments with new products lends support to the observations of social scientists who have studied young people’s inhaling habits (Nichter 2015; Dennis 2006).

Mimi Nichter (2015), in an elaborate ethnographic study conducted at a university campus in the United States shows that many students start to smoke for social reasons, and that they usually do so at social events such as parties or get-togethers. Smoking—used often together with alcohol—acts as a social lubricant, and it helps users communicate their emotions. Following more than 900 participants over time, Nichter found that students considered their smoking “no big deal” because they expect to quit when they leave campus and have a job. However, quitting often turned out to be harder than they expected. Some of her interlocutors transitioned to smoking regularly, including when they are alone, to reduce stress or overcome unpleasant life events such as relationship break ups.

We begin here by examining the evolution of smoking devices and regulations, and how these were attractive to Parisian youth, who started using e-cigarettes because they wanted to avoid the stigma of smoking ordinary cigarettes. And then we turn to a group of young smokers in Indonesia, who substituted cannabis with new kinds of synthetic cannabinoids, which they mixed with ordinary tobacco, to avoid being caught by the police ( cannabis smoke is easily detected because of its strong, recognizable smell). Being caught smoking cannabis in Indonesia is a serious crime that leads to imprisonment—possibly even the death penalty—while tobacco smoking is promoted heavily through advertising, and is highly acceptable (nearly the norm) for men. Our interlocutors in both settings sought to reduce harm, but they did so in different ways, and were encouraged in their efforts by different perceptions of risk. In this chapter, we also track the rising popularity of JUUL in US high schools among boys and girls who have not yet smoked regular cigarettes, and we show how JUUL hired young social media influencers to promote their product.

## New Ways of Inhaling Chemicals

There has been a surge in new inhalation devices in the past 20 years, which analysts suggest is related to the increased regulation of ordinary cigarettes (Russel 2019). In 2003 the World Health Organization’s Framework Convention on Tobacco Control, or FCTC, was adopted by 160 nation states. The treaty calls on signatories to regulate advertising and labeling of tobacco, increase the price of and taxation on cigarettes, and implement cessation programs. Faced by increasingly constrained markets for ordinary cigarettes, tobacco companies have embraced e-cigarettes as next-generation “ safer nicotine products” (SNP), which they market as a way to reduce the harm caused by cigarette smoke (Russel 2019). There are many different kinds of e-cigarettes. Some come in the form of pens or pipes, while others look more like cigarettes or USB sticks. “ Open” vaping systems can be used to vaporize any kind of fluid, while others, such as JUUL, are designed for specific liquid cartridges or dry material pods. The liquids and pods may contain nicotine as well as flavorings, and other ingredients.

Recently, the legalization of cannabis in Canada and parts of the United States have led to new lines of e-cigarettes. Young people now inhale marijuana not from “joints” but from novel devices and cartridges filled with cannabis extracts and flavored oils. The *New York Times* reports an estimate that vaping products account for 30% or more of the business of the legal cannabis industry, pointing out that the products attractive alternatives to joints, because they don’t produce ash, are easy to hide, and don’t have a strong smell (Richtel 2019).

**Fig. 3.1:**
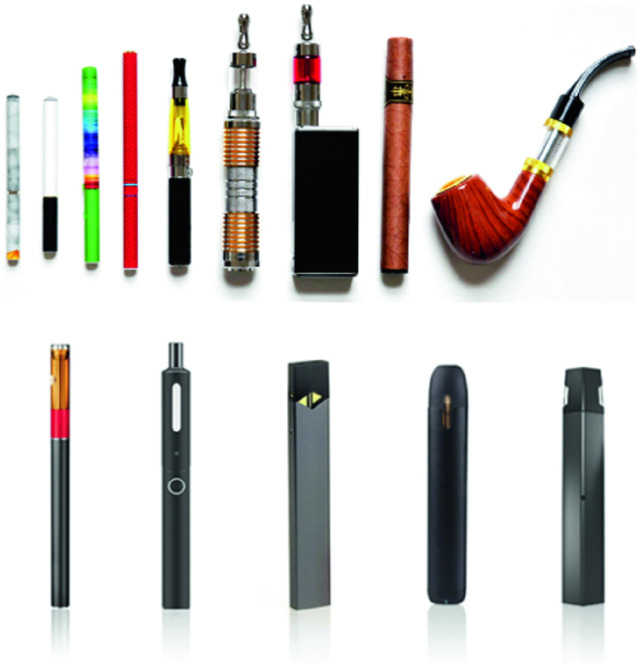
Screenshot of different kinds of e-cigarettes (*Source* Photo taken by Anita Hardon, January 2020, the Netherlands, US Food and Drug Admininstration (FDA) [2020])

Data from the National Youth Tobacco Survey 2016 show that in the United States among e-cigarette users aged 9–19 years, 33% of boys and 27% of girls had vaped cannabis (Trivers et al. 2018). That so many young people have inhaled vaporized cannabis is not surprising, given that media expressions tend to emphasize positive effects of the products: an analysis of YouTube videos portraying the vaping of marijuana found that only 2% of media expressions noted the potential for harm, while 68% pointed to positive effects (Yang et al. 2018) (Fig. 3.1).

## The Turn to Vaping in Paris

Imogen Bevan did a focused ethnography of vaping in Paris, in the same year that JUUL was designed by the Stanford graduate students. At the time of her fieldwork, a leading e-cigarette company in France was Gaiatrend, whose founder, Didier Martzel, like the JUUL inventors, declared that he had a harm reduction aim: to stop his sons from smoking tobacco. According to the company’s website, it intends “to offer smokers a completely new alternative and a solution to stop smoking on a long-term basis while responding to a real challenge in terms of public health” (Gaiatrend 2020).

The efficacy and safety of e-cigarettes as a way to quit smoking was heavily contested in French regulatory and health circles. France’s Public Health Council (Haute Autorite de Sante 2014) has focused on companies attracting youth into unhealthy nicotine consumption by means of e-cigarettes, while a study by the French Monitoring Center for Drugs and Addiction (Observations Francaise des Drogues et des Toxicomanies 2014) suggested (as JUUL’s designers emphasize) that e-cigarettes may be a “way out of smoking” (p. 8). In late 2014, Minister of Health Marisol Touraine declared that anything that can help smokers to stop smoking is worth taking. Even so, the government announced a ban on vaping on public transport, in offices, and in schools (Bevan 2016; Boudet 2014). Joining the chorus in 2015, a UK government health committee estimated that e-cigarettes are 95% safer than smoking (Public Health England 2015; see also Britton et al. 2014).

**Fig. 3.2:**
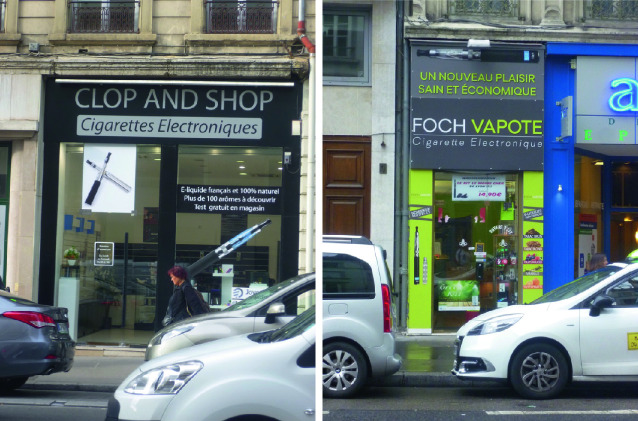
Examples of dedicated vaping shops (*Source* Photos taken by Imogen Bevan, September 2015, Paris. Note that the Clop and Shop vendor [left] offers 100 aromas and that the Foch Vapote shop [right] uses the tagline “*un nouveau plaisir sain et economique*” [a new pleasure, healthy and economical])

Many of Bevan’s interlocutors said they used e-cigarettes as a way to smoke less conventional cigarettes, which they kept for “special” moments. Using this strategy, they hoped to prevent the accumulation of tar in their lungs. Vendors in dedicated shops played a key role in the promotion of e-cigarettes and other substitutes for ordinary cigarettes by offering a wide range of attractive flavors (Bevan 2016) (Fig. 3.2).

The shops generally use a loyalty system that rewards clients with “gifts” (generally e-liquids) and retains a buyer’s history. The vendors advised customers to first replace cigarettes with e-cigarettes, and then to gradually lower the amount of nicotine they were consuming. The vendors calculated the nicotine consumed via ordinary cigarettes (nicotine volume multiplied by the number of cigarettes smoked per day), and advised consumers how much nicotine was contained in the available e-cigarettes. Bevan’s young informants were well aware of the dangers of smoking, and even before they switched to e-cigarettes, they did all kinds of things to reduce risks, including using disinfectants to remove carbon monoxide from their hands and switching to organic tobacco. In this sense, e-cigarettes were a welcome replacement device for what were perceived as risky and toxic cigarettes (Bevan 2016).

Bevan observes that, at least in Paris, taste was a key driver of vaping. Young people who smoked e-cigarettes compare their hobby to wine tasting and called themselves “flavor junkies”. Social gatherings where young people vaped together evolved into spaces of sensory exploration, as users passed e-liquids from hand to hand and shared experiences and tastes—the shared “gustatory pleasure” fostered feelings of community (Bevan 2016).

Bevan (2016) points to the emergence of vaping support groups and social media sites for e-cigarette users, where people share their experiences and promote products. The forums share more than an intention to stop smoking: they help vapers learn how to use e-cigarette devices (McQueen et al. 2011). Members of such groups regularly come together to explore new possibilities and enjoy their practice. The homepage of one vaping forum reads: “We are a big family. We intend to provide continuous support, from the discovery of e-cigarettes to the evolution of confirmed vapoteurs” (Bevan 2016, p. 237). Users’ profiles on the forum identify their status, such as “beginner” or “expert.” Members bear witness on the sites, enumerate how many toxic ordinary cigarettes they have avoided since they started vaping, and declare their vaping history in great detail.

In these vaping forums, users discuss a variety of issues, including technical issues related to the devices, such as a leaking or dead device, and laws and regulations. But conversations also include more personal concerns, such as having problems with one’s partner or wanting to lose weight. Health warnings are posted by members when news emerges on adverse effects of specific kinds of liquids or devices. One section of one of the forums announces in-person gatherings, called “vapodays,” and information about where they are to be held. Bevan describes a July “vapnique” (a combination of “ vape” and “picnic”) she observed in a city park in Paris:

On one side of a tartan picnic rug dappled in sunlight, a man asks quietly around him whether anyone else had changed weight when they had stopped smoking. He had put on 5 kg. What is he on? One man asks. Pausing to draw on an intricate wooden e-cigarette and exhaling a large cloud of apple and cinnamon vapor, he replies: 0 mg of nicotine. “It’s the nicotine, it makes you burn calories. It affects the brain,” a second man ventures. Well, he had had nicotine in it before and it made absolutely no difference, he was putting on weight anyway. The four of us shift position and recline to enjoy the now bearable late afternoon sunshine, slowly absorbing each other’s smoking histories, tales of transition and experience of e-cigarettes.

Seated nearby in a circle in the shade, a larger group is leaning over a pile of small bottles of different colors, shapes and sizes. A woman in her forties is interested in how long e-liquids should be kept. The younger woman on her right answers “Officially, a year, but I think you can keep them a bit longer than that.” While passing around a plastic tub overflowing with homemade brownies, the group goes on to discuss their preferred brands of e-liquid and to question the traceability of e-liquid contents. The five members converge on a general aversion to Chinese liquids as lacking sufficient safety regulations. (Bevan 2016, p. 238)

What stands out in Bevan’s ethnography is that one of the attractions of e-cigarettes was the social bonding that comes with the devices, bonding that was facilitated through social media. E-cigarettes allowed youth to experiment with different dosages of nicotine and align their vaping tastes to specific social settings and particular foods. Jem, a 25-year-old employee in a sports shop, explained that he had really suffered when he tried to quit smoking:

There was something missing from my life. And it’s funny, I felt like I wasn’t the same person anymore. I was sharing a flat with a friend, and strangely, when I stopped smoking, our relationship was a bit different. It was a bit tenser. Since I wasn’t smoking, we couldn’t share that moment together anymore. For me, smoking is a kind of sharing. When I had friends who quit, I was—I didn’t like it. I felt like I had lost something we had in common. (Bevan 2016, p. 237)

An analysis by Euromonitor (2019) notes that the higher costs of cigarettes due to higher taxes (as proposed in the FCTC) in France encouraged users to adopt vaping products, which were much cheaper. Statistics show that prevalence of smoking among men is gradually declining in France: 40% of adult males smoked in 2000, and this dropped to 35% in 2016 (World Data Bank 2020). Rates for women have stayed the same. A survey conducted in 2019 found that 6% of adults vaped regularly, with health, wanting to quit smoking, wanting to find an alternative to tobacco, and cost listed as the most important reasons to vape (Statista 2019).

## Inhaling Sinte in Indonesia

**Fig. 3.3:**
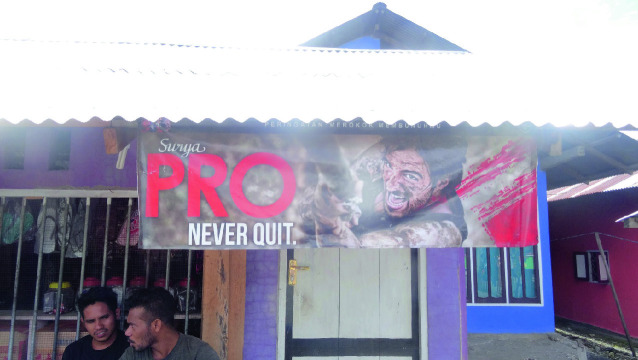
Cigarette banner (*Source* Photo taken by Anita Hardon, December 2018, Indonesia)

Indonesia is one of the countries that has not signed the FCTC. Cigarettes are extremely cheap throughout the country and advertising is omnipresent and aggressive. Not surprisingly, e-cigarettes are not popular in the country. On weekends and holidays, young girls in sexy outfits offer people in bars and restaurants free samples of cigarettes. The country is home to many tobacco companies that cater to the local market, producing cigarettes that contain a combination of tobacco and cloves ( *kretek*). The lack of tobacco control policy is reflected in increasing smoking rates among men. In 2000, a reported 60% of adult men smoked, while in 2016 this percentage was up to 76%; in contrast only about 3% of women smoke in the country (World Data Bank 2020). It’s almost impossible to not see tobacco advertisements in Indonesia given that most grocery stores have a banner sponsored by a cigarette company, see below picture taken during a field visit to the remote island of Morotai (Fig. 3.3).

Gudang Garam, the company that products Surya PRO, chose “NEVER QUIT” as its tagline. An analysis of advertising conducted by Mimi Nichter and colleagues (2009) shows how manufacturers present smoking as a way to relax, control emotions, and enjoy social gatherings. As is the case for JUUL marketing in the United States, youth are an important target for advertisers. Marlboro and Star Mild use advertisements designed to attract young men, emphasizing masculinity and sports. A Class Mild advertisement targets young women who want to be modern, with an image showing a young woman with a cell phone wearing a sleeveless top, which “is noteworthy in a country where women are expected to dress modestly and may wear headscarves” (Nichter et al. 2009, p. 103). The text, written in English, reads: “Yesterday is gone. Class Mild is today” (Nichter et al. 2009, p. 102).

In a fascinating historiography of tobacco in China, Matthew Kohrman (2018) argues that tobacco companies strategically “go with the times” in the images they use to promote smoking. Visuals during the reign of Chairman Mao showed men smoking only during political study and work; women were not portrayed with cigarettes, as it was seen to be synonymous with bourgeois decadence (Benedict 2018). China, like Indonesia, has a powerful state-run tobacco industry, and while China has signed the FCTC it is not taking strong measures to implement the treaty (Kohrman 2018).

In Indonesia, cigarette smoking is ubiquitous among men, who often also smoke cannabis. One of the ChemicalYouth ethnographers, Akbar Alamsyah, described how a group of students from the University of Makassar often met to smoke cannabis together. Most of them started using cannabis out of curiosity, when they were still in high school. They touted its relaxing effects, while also pointing to its creative potentials. They consumed it mainly when hanging out with friends, and when they needed to, they pooled their money to buy it. Because it is a plant, they considered cannabis to be harmless and natural—a valuation that meshed with their experience that, while the substance caused them to feel mellow and relaxed, they didn’t “go out” (black out, pass out, become unconscious) completely, as has happened with some of the other substances they have used. Some informants, though, said that they had experienced emotional turbulence and even paranoia when smoking cannabis. The effects, they explained, depend on whether the substance is “ *cocok*” (compatible) with you.

These interlocutors insisted that they really did not understand why cannabis was included in the list of narcotics in Indonesia, and they complained that heavy policing had driven up the price and that smoking cannabis was now more dangerous (in terms of legal repercussions) than ever. They tried to conceal their use by blending cannabis with tobacco from commercial cigarettes, but feared being caught because of cannabis’s strong odor. For this reason, students used synthetic cannabis (referred to as “ *sinte*” in Makassar), which could easily be bought via Twitter or Instagram; just type the hashtag “#tembakausuper” (#supertobacco) into Instagram and you will see more than 100,000 posts from users and vendors.

**Fig. 3.4:**
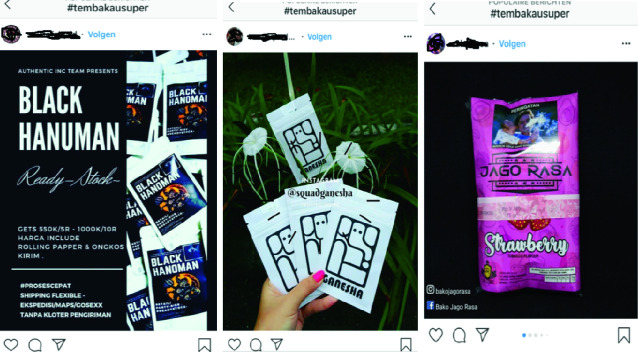
Screenshots of different kinds of Sinte advertised through Twitter and Instagram (*Source* Photo taken by Anita Hardon, January 2018, Indonesia)

Like Bevan’s respondents in France, our informants in Indonesia enjoyed trying out different products with other users. But they also observed serious adverse effects of these synthetic products. Sinte is sold under various names: Hanuman, Ganesha, Temchin, Nataraja, and Cap Gorilla (the last allegedly making users crazy as a gorilla, which the users found humorous) (Fig. 3.4).

Our interlocutors explained that *sinte* contains ordinary tobacco (and sometimes other plant fibers, such as tea) and synthetic cannabis (that is sprayed on the plant material). Sometimes synthetic flavors (such as banana, strawberry, or chocolate) are added, which add to its attraction.

The first time Basaka, one of our key informants, used *sinte*, he was 18 years old and still in high school. He was watching basketball in a local park and his friend offered him a puff of what he thought was ordinary ganja (slang for marijuana). After the first puff, he asked his friend, “Why is it like this?” His friend replied that it was Temchin (a *sinte* imported from China). Bakasa really liked the effect: it worked faster than ordinary ganja.

Our interlocutors emphasized that you can recognize a *sinte* user because his eyes are red and his lower eyelids are puffy. They said that the benefits of *sinte* are that it makes you feel relaxed and that you have fun with your friends. Another advantage is that is does not smell like ganja, so it is safer to use in public. But *sinte* also made some users feel nervous and they found it hard to communicate. As well, they could become thin, because it made them too lazy to prepare food and eat.

Usually Basaka and his friends smoked *sinte* in the afternoons and evenings, when they have finished school or work, to relax and enjoy time together. It made them feel refreshed, even more so when they watched the sunset. The locations where they gathered to use *sinte* were relatively private spaces, such as rooms in their homes, cars, rooftops, and islands (Makassar is a harbor town, with easy access to many small and isolated islands that are secure places to smoke, the breeze making it even more pleasant). Basaka hadn’t told his family members that he smoked *sinte*, as he was afraid they might judge him or confiscate his supply. He was also afraid of being arrested by the police. He did, however, confide in his niece, who he could tell by her reddish eyes, also was using *sinte*. She was also afraid of being arrested by the police.

Our interlocutors bought *sinte* from dealers they contacted via text message. In their communications, they used code words, for example asking “*ada bahanmu*?” (do you have stuff). They met the dealers in their homes, in the homes of friends, or on the street; this often involved taking some puffs together to gain the dealer’s trust. The process of ordering and consuming was also referred to as “having a snack.” The costs of *sinte* varied from 100,000 to 350,000 IRP (US $7–25) for a sachet, which can be used for six or so cigarettes—usually our interlocutors blended it into pre-rolled cigarettes to not make it look like a joint. Unlike ordinary cigarettes, *sinte* was shared, making it affordable for a group of friends. Basaka also said that he had bought it in bulk, making small sachets himself to make some profit to finance his own use.

Mixing *sinte* into cigarettes is a craft, one our interlocutors enjoyed doing. They tinkered with dosages to create the optimal effect. They each had their own preferred brands of cigarette tobacco with which they cut the *sinte*. Basaka preferred red of white Marlboros. Sampoerna (a *kretek* cigarette), which others used, was too sweet, he said. Some of his friend used menthol cigarettes as a base. To remake the cigarettes, after mixing tobacco with *sinte*, our interlocutors used rolling paper, which also came in different brands (and at different prices). Raw paper was more expensive, as it is made out of cannabis fibers. Marsbrand was cheaper, and therefore used more often. Sometimes the youth simply reused the paper of the cigarettes from which they took the tobacco. To these crafted joints they added a cigarette filter, to make inhaling easier. Or they rolled up tax-stamps found on cigarette boxes to make a filter. Others used pens or water bottles to help inhale the tobacco.

Bakasi said he was tapering his use. He would smoke a joint if invited to by a friend, because smoking joints together was highly valued. But overall, he tried to avoid *sinte*. He explained, “I want to keep having fun, but … it’s not good for my future.”

Karina Maharani (2017), a writer for the online news forum Rappler, reported how a 24-year-old tech worker she calls “Jay” used *sinte* in Jakarta:

The gold-foiled packet that Jay holds up is small—about 10×10 cm—and innocuous-looking. You could stow it easily in your pocket, or your wallet. When opened, the contents seem similarly harmless. To the naked eye it looks just like ordinary tobacco, a familiar sight in a country of 61 million smokers. He takes a pinch or two from the packet and puts it on rolling paper. The resulting cigarette looks pretty much like a joint of marijuana. Sitting cross-legged on the floor of a dim apartment in Jakarta, he lights it up and takes a drag, and then another one. The effects are almost instantaneous, within a minute. His hands tremble, his eyes redden, and his body slackens. “Two drags are enough to get high,” he murmurs, his eyes half-closed. “Your head feels heavy and your body feels limp.” The high won’t last long, Jay says, maybe 20 or 30 minutes. He doesn’t want to take another drag, because he has seen what overuse looks like. “A friend of mine, it was as if he was possessed by a demon.” Convulsions, hallucinations, yelling. Jay’s friend … 30, says he experienced something similar when he had too much—7 drags to be exact. “I felt like I was possessed,” he says. “I started getting very aggressive, like I wanted to fight everyone.” … “When you smoke weed the effect is stable,” he says. “This synthetic stuff is not stable. It’s weird.”

“When I smoke this, I feel like I’m being brought very high, and then not long after, I fall back down.”

As a regular drug user, Jay says he’d prefer marijuana, which he says is cheaper and “healthier,” but that he buys super tobacco because of its convenience (“It’s so easy to get”) and its efficiency (“The high is so much higher”). Dwi, a marketing professional, holds up Jay’s unfinished joint. “Can I light this spliff first?” he asks. “To make it more soulful.” He takes a drag before speaking to us. Dwi, who like Jay uses drugs regularly, waxes a little poetic about marijuana, calling it God’s creation and comparing it unfavorably to the synthetic version.

“You can be creative on weed, productive,” he says. “On this stuff,” he holds up the joint. “You’re just a consumer.” He adds that his heavy user friends refuse to smoke super tobacco because “it’s always a bad trip.” Like Jay, Dwi says that if he could choose between marijuana and super tobacco, he would pick the former. But these days with the marijuana supply dwindling, he’s smoking more of the latter, mainly because it’s so easy to get. “It’s just like ordering fast food.” (Maharani 2017)

Indonesian *sinte* smokers are concerned about adverse effects, which is to be expected, according to Michael White, a professor of pharmacy at the University of Connecticut, who explains:

There are several hundred synthetic cannabinoids in existence, and they all stimulate cannabinoid type 1 receptors (CB1), just like the active component in natural marijuana, THC, that provides the high. But they do so with different intensities and for differing periods of time. There is no way to know which synthetic cannabinoids are actually in the product you purchase. (White 2018)

White explains further that natural cannabis not only contains THC but also has cannabidiol, which actually helps to temper the negative impact of THC. Synthetic cannabinoids do not contain cannabidiol. Moreover, they are often mixed with other chemicals, ranging from opioids to rat poison, which makes their effects even more unpredictable. It is unjust that, in an era when natural cannabis is being legalized in so many places in the world, our interlocutors in Indonesia were turning to more dangerous substitutes from unknown sources.

Our ethnographic research suggests that the War on Drugs in Indonesia actually fueled the use of synthetic cannabis in Jakarta. Super tobacco was easy to get and was seen as safer by the young people who used it as a form of harm reduction. But, as the above observations suggest, adverse effects can be severe. Users cannot tell from the outside how much or what kind of synthetic chemicals have been sprayed onto the tobacco.

In 2019, Indonesian drug authorities made 25 synthetic cannabis compounds illegal (scheduled as Class 1 Narcotics), which means that drug enforcers are now free to crack down on the use and sale of synthetic cannabis. But our fieldwork suggests there will remain a high demand and continuous supply of new kinds of *sinte*, which given the informal networks through which the products are distributed, will be hard for authorities to control.

## Meanwhile, in the USA

**Fig. 3.5:**
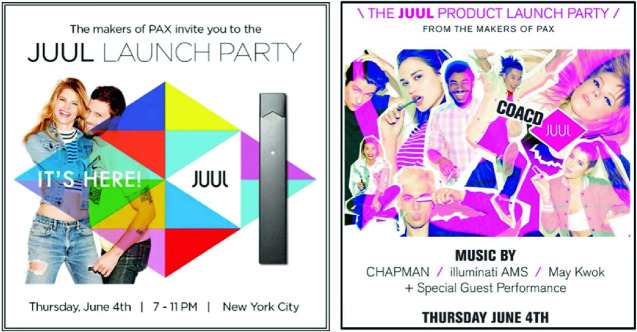
Youth-oriented launch parties (*Source* Photos taken from Jackler et al. [2019, p. 4])

While quitting smoking appears to be an important reason to start vaping in France, in the United States, concern arose about the popularity of JUUL among youth who had never smoked before. A research project entitled “Stanford Research into the Impact of Tobacco Advertising,” based at the Stanford University School of Medicine, connected the surge in the e-cigarette’s popularity to JUUL’s marketing strategy, which was emphatically youth oriented. Its “Vaporized” campaign, which started in 2015, featured male and female models in their 20’s in casual dress, along with bright colors. The researchers note that “the central message seems to be that if you try JUUL you will be blown away (i.e., vaporized) by the wonderful new vapor product” (Jackler et al. 2019, p. 7). Embedded in the campaign was the slogan “smoking evolved.” Along with this media campaign, the company offered free samples of JUUL flavors at youth-oriented music events and movie nights (Fig. 3.5).

The Stanford researchers describe how JUUL strategically sought out social media influencers as brand ambassadors, creating several Twitter hashtags, such as #Juul and #juulvapor, to promote the products. This social approach appears to be one of the key reasons for the popularity of the devices: young people like exchanging their experiences with each other.

**Fig. 3.6:**
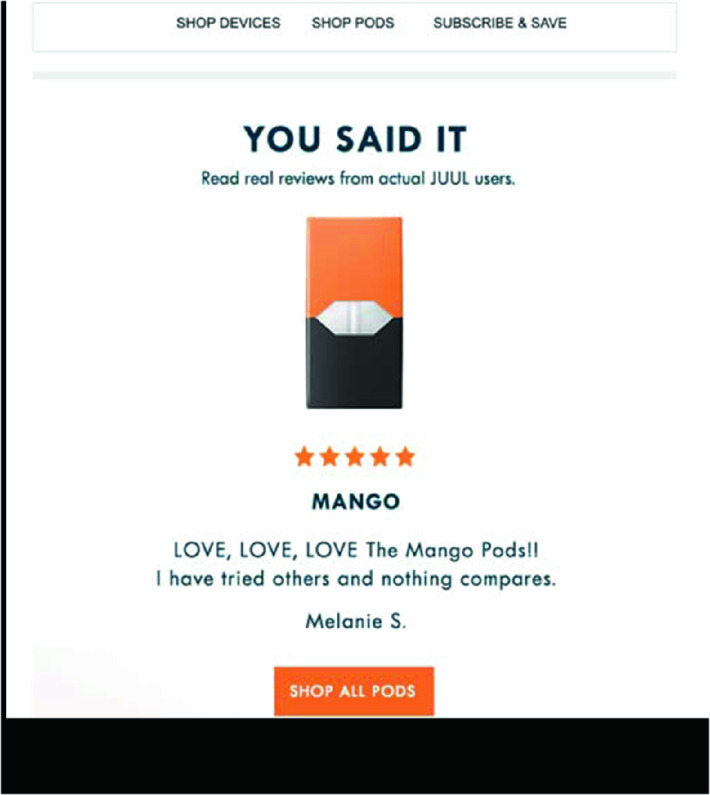
This 2018 advertisement was part of a JUUL promotion for mango pods (*Source* Photo taken from Jackler et al. [2019, p. 11])

Since its inception, flavors have also played a key role in the marketing of JUUL, with mango being the most frequently tweeted flavor. Figure 3.6 shows a 2018 JUUL advertisement for this flavor. Note how the advertisement cites users, with “YOU SAID IT” in capital letters, and then a quote, like this one from Melanie S., reading “LOVE, LOVE, LOVE the Mango Pods!!”

Jackler and colleagues (2019) also observe that, during its launch in 2015 and early 2016, JUUL did not warn young people about the nicotine content of its pods. This was a glaring omission given the considerable nicotine content of JUUL pod, each of which lasts around 200 puffs and contains 40–59 mg of nicotine, which is equal to the nicotine content of an entire pack of cigarettes. This marketing strategy is also very different from that chosen by Gaiatrend in France, where slowly reducing nicotine levels through company-provided software was one of the selling points. The Stanford medical researchers conclude that the company’s declared aim of “improving the lives of the world’s one billion adult smokers” did not align with its actual marketing practices. Two rationales—switching from ordinary cigarettes to e-cigarettes, and adult-use only—became prominent themes in advertisements after JUUL’s youth-oriented marketing campaign was criticized by US regulators and parent groups, as well as several class action lawsuits (Jackler et al. 2019).

In November 2018, the US Centers for Disease Control and Prevention reported that current e-cigarette use among American high school students had reached 20.8%, representing a 78% increase from 2017 (Cullen et al. 2018). A 2018 survey among 14,379 teens and young adults (15–34 years old) revealed that use of JUUL was three times higher in teenagers than in young adults: 9.5% for teens aged 15–17, but only 3.2% for those aged 25–34 (Cullen et al. 2018). The CDC researchers found that the appeal of JUUL was threefold: the device’s resemblance to a USB stick, making it easily and discreetly used without parents and teachers noticing; its character as technological innovation; and its youth-friendly flavors, including mango, mint, crème brulee, and fruit medley (Ramamurthi et al. 2019).

## Unrecognized Harm

Our interlocutors in France and Indonesia shared with their friends their lived experiences with the products that they inhaled, including adverse events. They tinkered with dosages and flavors to have optimal experiences in “do-it-yourself” communities. Marketing *sinte* is illegal in Indonesia, and manufacturers do not advertise the products much, other than sharing how and where they can be obtained and how much they cost. Users posted images of the products on their social networks, but information on how to use and craft *sinte* cigarettes occurred in face-to-face gatherings.

The positioning of e-cigarettes as safe alternatives is fueled by the big tobacco companies, who see a new opportunity to sell nicotine (Elam 2015). Big Tobacco’s interest is evident from the establishment of the Foundation for a Smoke-Free World (FSFW) in 2017. The large tobacco company Philip Morris International funded FSFW with a substantial grant of US $80 million. In a press release celebrating this event, Philip Morris International (2020) announced that it intended to “build” its “future on smoke-free products that are a much better choice than cigarette smoking.”

The FSFW appointed Derek Yach, who had formerly directed the Tobacco Free Initiative at WHO, as its first CEO. One of FSFW’s first actions was to commission a study entitled the “Global Status of Tobacco Harm Reduction 2018.” This report categorizes e-cigarettes as “ safer nicotine products” (SNP), arguing that to encourage people to quit smoking:

the new devices need to be easy to use and cost-effective, but must also offer choice. These days, choice is what people expect—of beer in a pub, coffee in cafes, or butter in the supermarket. And just as design is critical to the marketing of smart phones, wearables and similar technologies, the design of new nicotine devices is important. SNP design needs to fit into the modern technological zeitgeist; indeed, the look and feel of many SNP is light years away from dried leaves wrapped in bits of paper. (Shapiro 2018, p. 23)

The report reviews evidence on potential harm from SNP and concludes that public health researchers and medical professionals who warn against e-cigarettes mislead the public by adhering to the precautionary principle. The authors argue that that position is misguided, because “there is a potentially huge saving in mortality and morbidity to be made by actively encouraging persistent smokers to switch to SNP,” and that there is a danger from the “global drive to over-regulation and control” (Shapiro 2018, p. 77).

While e-cigarette manufacturers (and government policy makers) suggest that vaping is a safer administration route, given it avoids the tar and other toxins associated with the burning of tobacco leaves, many medical specialists have raised concern about the safety of the adjuvants and flavors included in e-cigarette liquids and pods. A 2014 contribution to the *Journal of the American Medical Association* argues that flavorings are “a largely unrecognized potential hazard” (Barrington-Trimis et al. 2014, p. 2493). The flavors have been mainly evaluated for their safety in food, which is ingested, and such evidence is thus not relevant to the inhaling of the substances. They point to the risk of irreversible obstructive lung disease associated with diacetyl (Kreiss 2014), which was found in 69% of sweet-flavored vaping solutions examined in a recent study (Farsalinos et al. 2015).

As of the writing of this chapter in 2020, five years after the early warnings of harms associated with vaping chemicals were reported, JUUL is front-page news following an outbreak of severe lung illnesses which has left 1479 people sick and 33 dead. The health authorities found that the e-cigarettes that caused the illnesses contained THC (Ritchel 2019), and that the ingredient linked to the lung disease appears to be vitamin E acetate (which is used as a filter in THC containing e-cigarettes). Until more information is known, the US National Institute on Drug Abuse have warned young people not to vape THC containing products (NIDA 2020a). In September 2019, the US FDA sent a warning to JUUL to urge them not to market e-cigarettes as a safe alternative to ordinary cigarettes. A few days later President Trump said he planned to withdraw flavored e-cigarettes from the market and in October 2019 JUUL announced that it would stop selling flavored vaping pods in the United States. In February 2020, the FDA followed suit and banned all flavors except menthol and tobacco in pod-based e-cigarettes. However, the ban doesn’t cover e-liquids, which youth can use as alternative to JUUL (Sindelar 2020).

Recognizing these problems, JUUL’s chief executive, K.C. Crosthwaite, said in a statement, “We must reset the vapor category by earning the trust of society and working cooperatively with regulators, policymakers, and stakeholders to combat underage use, while providing an alternative to adults smokers” (Wells 2017, p. 1). At the same time, JUUL is gearing up to expand its market in Indonesia, using a US $12 billion investment from Altria, the manufacturer of Marlboro cigarettes, a powerful brand in the country (Tiku 2018). The company plans to sell JUUL in two popular grocery stores, Alfamart and Minimart, which can be found on every street corner in urban areas, ensuring that this brand of e-cigarettes will be more accessible than other brands sold mainly online and at dedicated vape shops.

In countries where cannabis is legal, vaping liquids are on the market and can be used in vaping devices. Faced with the uncertain content of the *sinte* products used by our respondents in Makassar, this would appear to be a safer alternative. Indeed, medical evidence suggests that vaporizing cannabis is safer than combustion, as the temperatures reached in vaping devices are lower, which means that fewer carcinogens and irritants are produced and inhaled (Earleywine and Barnwell 2007). However, in assessing safety, it is important to also consider the thinning agents added to e-cigarettes: propylene glycol and polyethylene glycol 400 (petroleum-based liquids) and vegetable glycerin (also called glycerol), a sugar derived from plant oils. Like flavors, these adjuvants are considered safe for use in the food industry, but when heated to vaporizing temperatures, carcinogenic substances such as formaldehyde are produced. Formaldehyde can also cause watering and burning eyes, and burning sensations in the throat (Troutt and DiDonato 2017).

## Changing Regulatory Regimes

In July 2019, the Philippines secretary for health, Francisco Duque III, followed the stance of the French minister of health in forbidding the use of e-cigarettes in public spaces. President Duterte, himself an ardent smoker, had banned cigarette smoking in public spaces two years earlier; taxes on cigarettes (in the category of “sin taxes”) are very high in the country (Philippines CNN 2019). In the Netherlands, a country without a substantial tobacco industry, the government is similarly committed to a smoke-free generation. Speaking at the 2018 biannual meeting of the World Health Organization Convention on Tobacco Control, Paul Blokhuis, the Netherlands’s state secretary for health, welfare, and sport, declared:

We have a clear goal: to raise a generation without tobacco—to ensure that children born today will never touch a cigarette… Our mission will require raising children in smoke-free environments. This means smoke-free homes, smoke-free schools, smoke-free public areas. If we want to prevent young people from taking up a smoking habit, we will need to help adults who smoke to quit. (World Health Organization 2018)

While Blokhuis’s compliance with the global treaty on tobacco control is commendable, he may appear to be missing an important new trend. A survey of a representative sample of 6718 youth in the Netherlands found that 36% of 16-year-old youth and 39% of 15-year-old youth had tried an e-cigarette (Utrecht University 2018). In 2017, Philip Morris Holland introduced an e-cigarette called IQOS, accompanied by an advertising campaign that claimed that the device has 90% less nicotine. And in 2018 the first JUUL pods entered the country (Euromonitor International 2019).

While global health policymakers have joined forces in the FCTC to reduce harm from nicotine addiction, our ethnographic research shows that many youth are engaging with these novel devices, which can be used as substitute for ordinary cigarettes. It is worrying that despite declaring a commitment to promoting e-cigarettes to adults, for the purpose of quitting ordinary cigarettes, companies are targeting youth through social media. It is also worrying that the products contain a wide variety of flavors and active ingredients, the safety of which has not been sufficiently studied, and that regulators appear to be taking for granted manufacturers’ claims that e-cigarettes are a safe alternative to smoking tobacco.

It seems a ripe time to apply the precautionary principle to this new chemical market, as also suggested by Gotts and colleagues (2019), who recently published a review of 193 studies in the *British Medical Journal*. They conclude that because no long-term research on toxicity has been done in humans, it is too early to say “with certainty” that e-cigarettes are safer. Their review found that e-cigarettes can cause increased symptoms of respiratory disease and negatively affect lung physiology and immune function. Gott, who is a pulmonologist at the University of California San Francisco, is quoted in *New York Magazine* as stating:

People are conducting a huge experiment on themselves about what kind of lung disease you can produce from all these different chemicals that you’re putting into the lungs. … And the bulk of the evidence is increasingly that these devices have new and unpredicted toxicity. (Hall 2020)

We propose that tobacco control programs start discouraging the use of e-cigarettes as a means to quit smoking, as there are better methods to quit smoking with proven success. We also propose that programs that seek to achieve a “smoke-free generation” expand their scope to discourage the use of e-cigarettes as well. This is even more urgent now the world is confronted with COVID-19, a viral disease that causes serious lung disease. The US NIDA warns youth “Now more than ever, it’s important to be smart about your health. Take care of your lungs: Avoid smoking or vaping any substance” (NIDA 2020b, p. 1).

Similar educational efforts are needed to discourage the use of *sinte* as an alternative to smoking cannabis, a message that is likely to be understood in Indonesia, as our interlocutors recognized the potency and health risks of substitution products. A challenge in such health education messages is that social media are full of positive accounts of vaping and *sinte*, which raises the question: How should we regulate online marketing of products to prevent harm? We think one way is to engage youth who act as influencers, and hope that they are willing to share cautionary tales.

## In Conclusion

This chapter provides insights into how young people seek substitutes to mitigate the risks of smoking cigarettes and cannabis. The harms that they seek to avoid differ. In the focused ethnography conducted in France, young people seek to avoid the risks of inhaling cancer-causing chemicals via cigarettes, while in Indonesia they fear being incarcerated because of being caught smoking cannabis. Harm reduction programs tend to overlook such substitution practices by youth. As we elaborate in Chapter 10.1007/978-3-030-57081-1_9, they tend to be designed to prevent harmful practices, one chemical at a time (Hardon and Hymans 2016). Failing to acknowledge the dynamic nature of young people’s chemical practices means that new risks remain under the radar.

Young people share their experiences with substitutes both face-to-face and online. Our ethnographies show how these interactions tend to amplify the beneficial potentials of the new products, especially when youth are encouraged to do so by manufacturers’ deliberate marketing strategy, as we illustrated in the case study on JUUL.

We describe in this chapter how tobacco companies “re-inform” (Barry 2005) their products into “ safer nicotine products,” in the form of e-cigarettes with a variety of chemical tastes and a diversity of inhaling devices.^1^ An epidemic of vaping-related lung disease occurred, which appears to have been caused by the chemical adjuvant used in the devices, an epidemic that could have been prevented if the precautionary principle—a cautious strategy of pausing and reviewing before allowing new chemicals on the market—had been adopted by governmental food and drug agencies for this new category of chemical device (MacKendrick 2018; Read and O’Riordan 2017). Young people value the new vaping technology because it allows for a continuation of social bonding through shared substance use (Duff 2004; Pilkington 2007; van Schipstal et al. 2016), the opportunity to experiment with new kinds of inhaling techniques and devices, and the opportunity to experience different flavors. They trust that the products that they can buy at their corner store are safe and this trust is further fostered by the face-to-face interactions through which information on the benefits of vaping are exchanged (Brown and Calnan 2012).

## ChemicalYouth Ethnographers

**Fig. 3.7:**
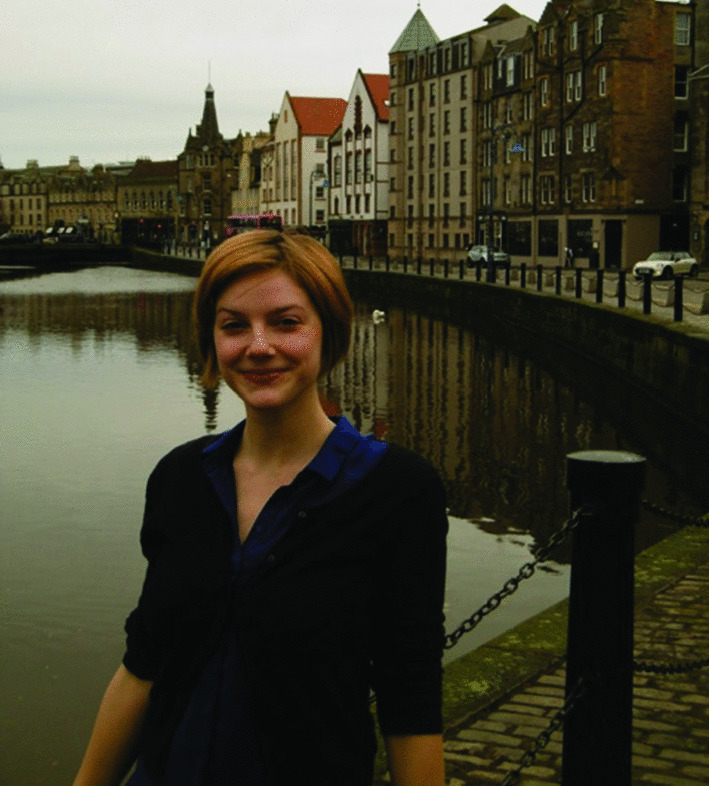
Imogen Bevan

Imogen Bevan is a social anthropologist at the University of Edinburgh. She has carried out ethnographic research in France on smoking and vaping, and in Scotland on sugar consumption and the social meanings of sugar. As a researcher for the ChemicalYouth project, Imogen examined young people’s lived experiences of smoking and e-cigarette use, and the socialities that emerge through non-medicalized forms of substitution. Her study used sensory and creative visual methods to explore what these technologies and substances might do for their users in social context. Imogen’s research interests include the anthropology of the body, morality, and kinship; the boundaries between food, drugs, and medicine; sensory anthropology; and visual methods (Fig. 3.7).

Akbar Alamsyah was a researcher for the ChemicalYouth project and conducted fieldwork on synthetic cannabis smoking practices amongst students in Indonesia.

### Note

1. In Chapter 10.1007/978-3-030-57081-1_1 we explain in detail what informing chemicals entails. We borrow this concept from Barry (2005) who argues that chemistry is a science of associations in which molecules are “informed.” We cite literature on how pharmaceutical companies reinform their blockbuster drugs to expand markets, and show how when young people appropriate chemicals in their everyday lives, they inform chemicals by producing shared knowledge on how best to use chemicals, and what chemical can do for them.
